# Cracking the Challenge of Antimicrobial Drug Resistance with CRISPR/Cas9, Nanotechnology and Other Strategies in ESKAPE Pathogens

**DOI:** 10.3390/microorganisms9050954

**Published:** 2021-04-29

**Authors:** Tanzeel Zohra, Muhammad Numan, Aamer Ikram, Muhammad Salman, Tariq Khan, Misbahud Din, Muhammad Salman, Ayesha Farooq, Afreenish Amir, Muhammad Ali

**Affiliations:** 1 Public Health Laboratories Division, National Institute of Health, Islamabad 45500, Pakistan; maahin1@yahoo.com (A.I.); salman14m@gmail.com (M.S.); ayesha.farooq@nih.org.pk (A.F.); afreenish.hassan@yahoo.com (A.A.); 2 Laboratory of Molecular Biology and Biotechnology, Environmental and Health Sciences, University of North Carolina, Greensboro, NC 27412, USA; m_numan@uncg.edu; 3 Department of Biotechnology, University of Malakand Chakdara Dir Lower, Chakdara 18000, Pakistan; tariqkhan@uom.edu.pk; 4 Department of Biotechnology, Quaid-i-Azam University Islamabad, Islamabad 45320, Pakistan; misbah@bs.qau.edu.pk (M.D.); alibiotech01@gmail.com (M.A.); 5 Department of Biotechnology, Sarhad University, Peshawar 24755, Pakistan; salmanbiotech55@gmail.com

**Keywords:** antimicrobial resistance, ESKAPE, bacteria, antibiotics

## Abstract

Antimicrobial resistance is mushrooming as a silent pandemic. It is considered among the most common priority areas identified by both national and international agencies. The global development of multidrug-resistant strains now threatens public health care improvement by introducing antibiotics against infectious agents. These strains are the product of both continuous evolution and unchecked antimicrobial usage (AMU). The ESKAPE pathogens (*Enterococcus faecium*, *Staphylococcus aureus*, *Klebsiella pneumoniae*, *Acinetobacter baumannii*, *Pseudomonas aeruginosa*, and *Enterobacter species*) are the leading cause of nosocomial infections throughout the world. Most of them are now multidrug-resistant, which pose significant challenges in clinical practice. Understanding these bacteria’s resistance mechanisms is crucial for developing novel antimicrobial agents or other alternative tools to fight against these pathogens. A mechanistic understanding of resistance in these pathogens would also help predict underlying or even unknown mechanisms of resistance of other emerging multidrug-resistant pathogens. Research and development to find better antibacterial drugs and research on tools like CRISPER-Cas9, vaccines, and nanoparticles for treatment of infections that can be further explored in the clinical practice health sector have recognized these alternatives as essential and highly effective tools to mitigate antimicrobial resistance. This review summarizes the known antimicrobial resistance mechanisms of ESKAPE pathogens and strategies for overcoming this resistance with an extensive overview of efforts made in this research area.

## 1. Introduction

In the mid-20th century, when the clinical practice of antimicrobial drugs was introduced, it revolutionized the public health sector [1]. The infectious microorganisms that had threatened human survival are now at the mercy of different chemical compounds. The introduction of antibiotics significantly reduced the risks linked with childbirth, injuries, and intrusive medical procedures [2]. On the other side, what has been observed in the last 70 years is ongoing microbial experimentation on a large scale and the haphazard use of antimicrobials in large amounts. This poses a genuine threat to human beings by pathogenic bacteria that acquire antimicrobial resistance. This alarms a coming time where common infections are as untreatable as in the pre-antimicrobial era [3]. It is assessed that by 2050, 10 million lives may be lost per year due to antimicrobial resistance. This exceeds the number currently lost due to cancer, 8.2 million lives [4]. To put this figure in perspective, every year, 700,000 people die globally due to acquired resistance against different antimicrobials, more than the total number of deaths caused by measles, cholera, and tetanus. The drivers of antimicrobial resistance are lesser knowledge about the best-applied practice of antibiotic stewardship and its education [5]; overuse of inappropriate antibiotics; unfair practices such as under or overdosing to treat minor bacterial, fungal, or viral infections; and most importantly the uncontrolled use of antibiotics in animal’s food to increase their meat production [6]. It is feared that if the current rise in antimicrobial resistance continues, the world economies will be hit by a loss of $100 trillion by the year 2050 [7]. As efforts are being made in research and development to find better antibacterial drugs, more research is performed in areas like CRISPER/Cas9, vaccines, and nanotechnology. The world health organization recognized these alternatives as essential and highly effective tools to mitigate antimicrobial resistance.

## 2. Drivers of Antimicrobial Resistance

During the 1960s, the first bacteria showing resistance to multiple drugs were *Shigella, Salmonella,* and *Escherichia coli* [8,9,10]. The increase in antimicrobial-resistant bacteria/pathogens poses a serious threat to the health sector and leads to extra-economic burdens. One of the significant contributors to this increasing antimicrobial use are the health care systems fighting against it, which allow inappropriate prescriptions and availability of antimicrobials without prescription to the patients, especially in developing countries. All this is then backed by the poor sanitation services, which aid the transmission, and low healthcare budgets have to rely on cheap antibiotics instead of the safer but more expensive ones [11].

**Figure 1:**
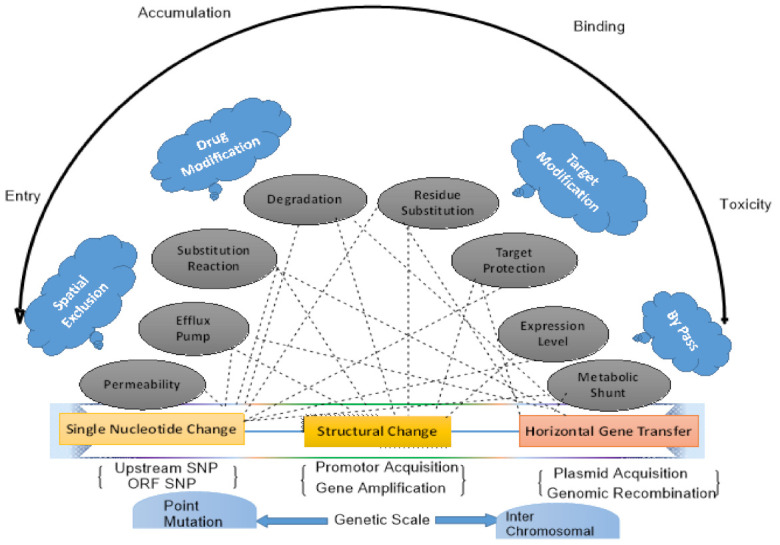
Genetic mechanism of Antimicrobial resistance in ESKAPE pathogen.

We are not creating antimicrobial resistance; we are simply endorsing it by putting on selective evolutionary pressure, which will result in the evolution of numerous genetic mechanisms [12]. Mechanisms by which antibiotics imply selective pressure are poorly understood. We have represented the genetic mechanism of antimicrobial resistance in the ESKAPE pathogen in Figure 1. Routes associated with antimicrobial resistance are dynamic and less predictable. Problems related to antimicrobial resistance can be assessed by simply recognizing two components: the antimicrobials that inhibit an organism’s susceptibility and the resistant genetic determinants in the microorganism selected by antimicrobials [13,14]. Subsequently, the resistance emerges when these two components interact in an environment or hosts, leading to several clinical problems. Over the years, constant evolution has led to the emergence of that *Enterobacteriaceae* strains, which have both MDR (multidrug-resistant) and XDR (extensively drug-resistant) strains [15], to nearly all antibiotics available, without any promising treatment alternatives [16].

Bacterial strains are tremendously effective vehicles to spread the antibiotic resistance traits, transferring them horizontally through mobile genetic elements (transposons and plasmids) or vertically to its daughter cells and other species [17]. These genes usually confer resistance against a single group or a family of antibiotics. A high level of resistance arises through sequential mutation in chromosomes, in the absence of plasmids and transposons, which typically mediate high-level resistance [18,19,20]. This scenario was the foremost reason for the initial emergence of penicillin and tetracycline resistance in *Neisseria gonorrhoeae*. Likewise, a group of *Enterobacteriaceae* acquired resistance to fluoroquinolones due to mutations in topoisomerase enzymes that alter gene expression and accelerate the membrane proteins that pump the drug out of the cell [18,20,21]. Resistant *Staphylococcus aureus* strains first appeared in response to vancomycin [22], followed by high-level resistant transposon from *Enterococci* [23,24]. An effective administration of contemporary antimicrobials, and the sustained development of the novel candidate, is crucial to protect human and animal health against bacterial pathogens [25].

## 3. Global Dissemination of Antibiotic Resistance

Several studies have been conducted on different samples of resistome from various environments, including studies of human and animal gut microflora, soil, and wastewater microbial communities [26,27]. Meanwhile, it has become clearly understood that ARGs (antimicrobial-resistant genes) related to clinical sides are prevalent in the environment [28]. Studies utilize metagenomics approaches to directly recover DNA from all microorganisms in a biological sample to investigate the resistome properly. Massive data has been generated from the sequencing of metagenomes and placed in databases. Such data will help in resolving different public health concerns. However, these studies’ data is only limited to identifying genes or predicting novel sequence-based on the same homology to the known reported sequence. Annotation by using sequence-based studies and functional genomics revealed the already known ARGs, which are prevailing in diverse conditions and environments such as in microflora of animals [29] and humans [30,31] in soil [32,33] as well as in activated sludge [34]. Numerous examples show that ARGs in human pathogens originated from soil and wastewater bacteria. One of the most well-known examples is blaCTX−M genes, which are the significant root of extended-spectrum b-lactamases (ESBLs) diaspora in *Enterobacteriaceae* globally and the main starting point of clinical treatment complications [35]. These genes’ marks were identified from chromosomal DNA of different conservational *Kluyvera* species found in soil and sewage. This can be the origin from where they are disseminated to diverse bacterial species [36]. Likewise, plasmid-encoded qnrA genes, presumed to be originated from fresh marine water species i.e., *Shewanella* algae, which confers Quinolone resistance, with its various *Vibrionaceae* species might also be considered as reservoirs [37]. This spread in different *Enterobacteriaceae* species globally in some areas with a high prevalent rate [38]. Even more, beta-lactamase genes, i.e., OXA-48-type carbapenem-hydrolyzing, progressively reported in various *Enterobacteriaceae* species, were also found to be originated from environmental *Shewanella* species [39]. It is thus believed that many clinically relevant resistance genes are found to be originated from non-pathogenic bacteria underlining the colossal potential of horizontal gene transfer (HGT) for these pathogens in overcoming human use of antibiotics.

## 4. Emerging Resistance–Development of Resistant Strains

**Figure 2:**
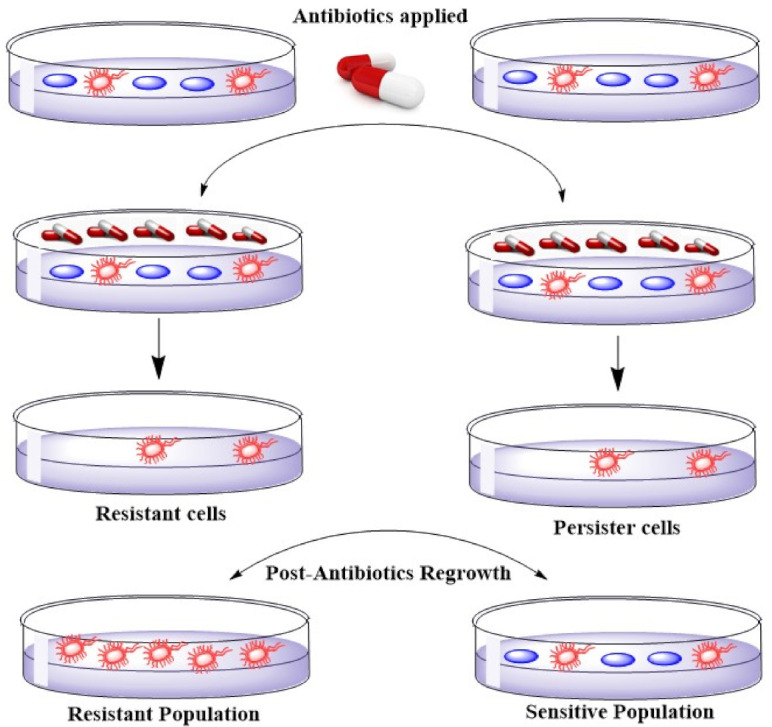
Illustration of the comparison of Resistance and Persistence in the bacterial population.

Resistance genes exist in association with genes specifying resistance to other antimicrobials on similar plasmids that lead to multiple drug resistance [40]. The occurrence of MDR plasmids assures the plasmid’s presence if any one of the resistances offers survival benefit to the host bacterium. This principle similarly implies every determining factor of resistance to biocides like quaternary ammonium compounds because plasmids bearing efflux genes exist that offer resistance to antibiotics in *S. aureus* [41]. Some studies show a decline in resistance frequencies when an antibiotic is removed [42]. A noteworthy coast-to-coast setback of macrolide resistance in *Streptococcus pyogenes* occasioned from a Finnish countrywide operation to reduce macrolide practice. In two years, the resistance dropped from about 20% to less than 10%. If a bacterium is resistant to a particular antimicrobial agent, then all the daughter cells would also be resistant (unless additional mutations occurred in the meantime). Persistence, however, describes bacterial cells that are not susceptible to the drug but do not possess resistance genes. The persistence is because some cells in a bacterial population may be in the stationary growth phase (dormant). Most antimicrobial agents do not affect cells that are not actively growing and dividing. These persister cells occur at around 1% in a culture in the stationary phase [43,44]. Figure 2 shows the difference between persistent and resistant bacterial cells. As depicted in Figure 2, persister cells tolerate the antibiotics by changing to a dormant state. These cells do not divide, and they develop tolerance to a high level of antibiotics. Unlike, resistant cells which develop resistance through accumulating mutations, tolerant persister cells are not antibiotic-resistant mutants. Antibiotic tolerance in persister cells is developed through going to a reversible physiological state in a small subpopulation of bacterial cells [45].

## 5. ESKAPE, Healthcare Concomitant Bugs–Bad Bugs with No Drugs

ESKAPE is an acronym for the group of pathogens, including Gram-positive and Gram-negative species, comprising *Enterococcus faecium, Staphylococcus aureus K. pneumoniae, Acinetobacter baumannii, P. aeruginosa, and Enterobacter species* (Table 1). The Infectious Disease Society of America has started referring to this group of hospital-originated pathogens as ESKAPE [46,47]. These bacteria are usually the reasons behind most life-threatening nosocomial infections amongst immunocompromised and critically ill individuals [7]. Klevens [48] revealed that around 1.7 million people are affected by hospital-acquired infections (HAIs) in the US hospitals, which are responsible for nearly 99,000 deaths each year. A survey of HAI in the United States (US) in 2011 reported a total of about 722,000 reported cases, with 75,000 deaths associated with nosocomial infections [11]. It has also been shown that hospitals using antibiotics are where drug-resistant strains first appeared [46]. For instance, *S. aureus*, which is known to be resistant to penicillin, threatened London’s civilian hospitals soon after the penicillin drug was introduced in the 1940s [7].

## 6. General Mechanism of Antimicrobial Resistance

**Figure 3:**
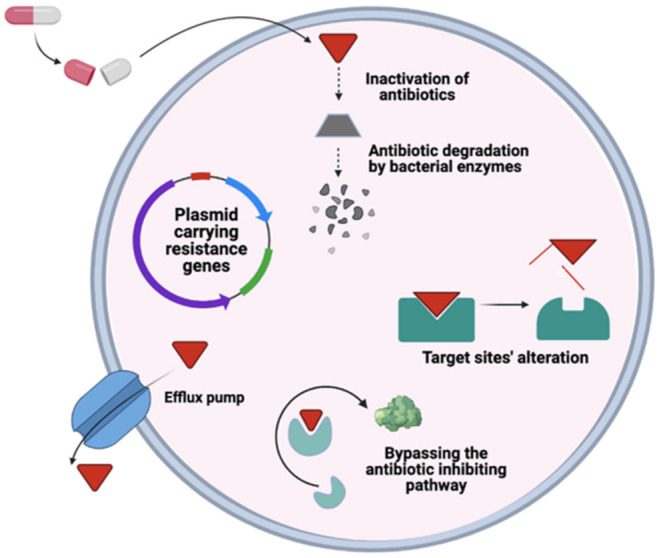
Illustration of the general mechanism of antimicrobial resistance in bacteria.

Many bacteria live as complex communities called biofilms in their natural habitat, including human hosts. These communities of bacteria offer enhanced resistance to environmental stress, including resistance to antibiotics [50]. The resistance that microorganisms obtain via biofilm formation can be approximately 1000 folds higher than the resistance obtained at the cellular level [50,51]. The development of resistance at a cellular level is endogenous gene mutations and horizontal gene transfer of resistance determinants through plasmids to other microbes (Figure 3). Apart from resistance, tolerance is also one way to evade antibiotics developed in persister cells, described previously [52]. Both types of resistance may be simultaneous, hence increasing the microbial community’s antimicrobial resistance [50] (Table 2).

## 7. Alternative Mechanisms for Combating Multidrug Resistance in ESKAPE Pathogens

### 7.1. CRISPR-Cas9

There are several applications of the cutting-edge technology known as Clustered Regularly Interspaced Short Palindromic Repeats and their associated Cas proteins (CRISPR/Cas system). As the CRISPR induces double-standard breaks, one could be the knocking out of a particular bacterial gene. This characteristic of CRISPR/Cas has led to its use to target specific genes for resistance located in plasmids. One of the advantages of using the CRISPR/Cas system is that it has the capability of multiplexing against different targets, which then enables it to target different resistance genes simultaneously. The question arises whether this approach can be effective in the removal of the resistant genes from MDR bacteria that are present in intestinal microbiota or not? The main limitation is to have a collection of appropriate temperate phages designed against multiple resistance genes, and that resistance genes carried by the bacteria should be known. This is feasible in the current situation. It has been observed that phages are well tolerated when they are orally administered [9]. The orally administered phase therapy for bacteria targeting present in the intestinal tract has been a success. However, to avoid bacteriophages’ deactivation by acid, the stomach must be passed before using the CRISPR/Cas approaches. However, there is a need to conduct further studies to confirm whether the phages will still be active then they reach the intestinal tract, and if not, how can we make sure of it? There is also a need to know the optimal dose that should be used.

Another advantage of this approach is that without compromising the patients’ normal microbiota, susceptibility to antibiotics is restored. Further development of the two approaches discussed above would be revolutionary in the fight against antimicrobial resistance. These techniques could be used for patients with MDR bacteria in various settings to prevent the spread of MDR bacterial strain [59]. On the other hand, the animals have also been shown to play an essential role in reservoirs of MDR bacteria. Therefore, these techniques can also be used for them.

### 7.2. Nanotechnology and Nanoparticles to Combat Multidrug Resistance

Several hypotheses have been put forwarded for the mechanism of nanoparticles of metals and metal oxides. The hypothesis includes protein dysfunction, physically disrupting the cell structure, generation of reactive oxygen species and depletion of antioxidants, impairing of membrane and interfering with the nutrient assimilation and use of dephosphorylation of the peptide substrates on tyrosine residues which help to alter the signal transduction resulting in its inhibition and suppressing the bacterial growth [60]. The nanoparticles derived from zinc oxide and silver can penetrate the bacterial cell wall and result in changes of its cell membrane, which causes structural damage; hence, the integrity of the membrane is lost, leading to cell death [61,62].

**Figure 4:**
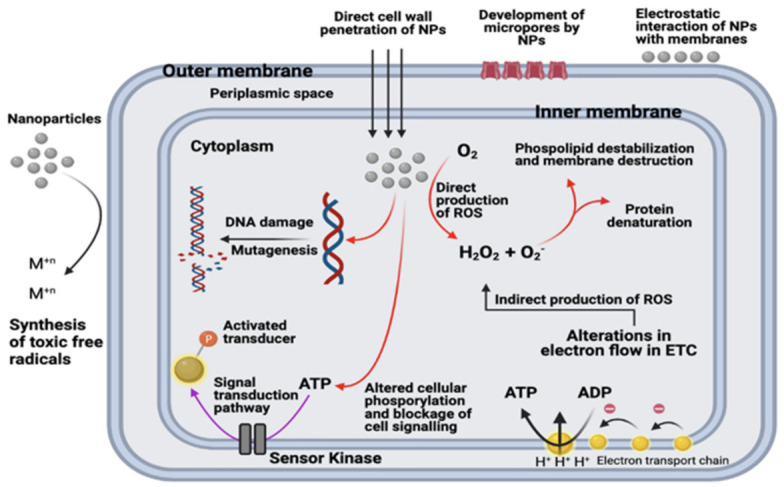
Suggested action mechanisms of metallic nanoparticles against gram-negative bacteria. Adopted from [67].

Silver nanoparticles are also known to mount on the cell wall and form pits in it, while gold nanoparticles apply their antibacterial activities by disintegrating the bacterial cell membrane [63]. Apart from these mechanisms, there is another mechanism in which free radicals are produced to generate oxidative stress. These generated reactive oxygen species can destroy the bacteria by destroying its DNA, membrane, and mitochondria, hence ultimately killing the bacterial cell [64]. However, there is a chance that the bacterial cells, to fight these reactive oxygen species, may produce more detoxification enzymes [65]. The metallic nanoparticles can interact with phosphorus and sulfur, present in biomaterials in bacterial cells like DNA bases. Hence, these can help destroy DNA resulting in killing the cell [66], (Table 3). Some of the possible action mechanisms of nanoparticle-induced death of bacteria are shown in Figure 4.

## 8. Host-Directed Therapies

### Promoting Bacterial Clearance through Modulating Host’s Inflammatory Responses Regulating PRR Signaling Pathways

To detect pathogen-associated molecular patterns, we can use pattern recognition receptors (PRRs). The known PRRs are RIG-I-like receptors, Toll-like receptors (TLRs), and NOD-like receptors (NLRs). A few germline-encoded pattern recognition receptors can identify a wide variety of molecular structures linked with the pathogens. Till now, a total of 13 Toll-like receptors have been found. The first nine of these receptors are reported to be conserved in humans and mice. However, it has been reported that humans do not express the other four TLRs. Studies with mice have revealed that each TLR has a different role in recognizing PAMP and the immune responses [85]. If we take an example of the TLR-2, it can heterodimerize with TLR-1 or TLR-6 in recognizing bacterial lipopeptides. TLR-9 can detect CpG islands that have an abundance of the bacterial genome, while TLR-4 can detect the presence of lipopolysaccharide. It has been seen that except TLR-3, all the TLRs activate a MyD88-mediated signaling cascade, which leads to nuclear factor *kappa*-light-chain-enhancer of B cells activation and upregulation of proinflammatory gene expression [86].

NOD-like receptors are also used to detect PAMPs present in cytosol, for example, NLR-P3 and NLR-C4. NLR-P3 is an inflammasome producing NLR. It involves the oligomerization of procaspase-1 through an adapter protein, the apoptosis-associated speck-like protein containing a CARD (ASC) [87]. Autoproteolytic cleavage of procaspase-1 results in its activation and can subsequently convert pro-interleukin-1 (pro-IL-1) and pro-IL-18 to their active forms [88]. Another molecule with the name of MCC950 was also discovered, which can inhibit NLR-P3-induced ASC oligomerization; however, it cannot work for NLR-C4 signaling activation [89]. Further studies are required to characterize the role of MCC950 in regulating bacterial infection. Identifying small molecules that can selectively prevent cytokine secretion upon NLR-P3 inflammasome activation appears to be a promising new therapeutic strategy.

## 9. Vaccine Development

Vaccines help train the immune system to identify and appropriately respond by generating a fast and effective defense against any pathogen, hence preventing disease/infection [90]. Some of the vaccines are also reported to protect the unvaccinated subjects, which is possible because of herd Immunity. Herd immunity is carried out for a large population. The population licensed to be vaccinated in the herd immunity is protected from the disease, but it also helps prevent transmission of pathogen/disease to unvaccinated subjects [91]. It is tested that herd immunity helps protects a much larger number of people than those who were vaccinated in the community. The studies show herd immunity’s success, for example, vaccines used against *S. pneumoniae* and Hib, which prevent pathogen colonization in vaccinated subjects.

One of the first vaccines that showed high effectiveness in preventing the disease and reduced antibiotic use was the *Haemophilus influenzae* type b (Hib) vaccine. It showed promising results in infants as well as older children by herd immunity. If we investigate the past before introducing the Hib conjugate vaccine in 1980, Hib was a very dangerous pathogen for infants and children. Hib cases at that time ranged from 3.5–601 cases per 0.1 million for children under the age of 5 years [92]. Due to the use of antibiotics during the 1970s, a rise in Hib β-lactam resistance was also noted mediated by bacterial expression of β-lactamases and, to a lesser extent, modified penicillin-binding proteins [93]. Another surveillance study was carried out globally during 1999 and 2000, and it showed that 16.6% of all Hib strains worldwide were β-lactamase positive. However, the numbers varied mainly from country to country [94]. Providentially, Hib conjugate vaccines’ discovery and proper deployment have turned the tide against antimicrobial resistance [95]. The early vaccines developed for Hib during the 1960s consisted of the Hib polysaccharide capsule conjugated to carrier proteins. It was reported that due to its introduction, the cases dropped rapidly [96].

Consequently, there was a reported decrease in the nasopharyngeal carriage as well, and this was a prerequisite for herd immunity and extended protection of unvaccinated populations. The numbers showed a significant decrease in cases after the introduction of a vaccine in 1980 from 2.6 cases per 100,000 (1986–1987) to 0.08 cases per 100,000 in (2011–2015) [26,27]. Similarly, within few years of vaccine introduction in the United Kingdom in 1992, the Hib disease in children less than five years was almost eliminated [97]. Reports also show that as soon as the vaccine was introduced, there was a significant drop in b-lactamase-positive strains [98].

Another disease reported to be leading the cause of serious illness in both adults and children worldwide is *S. pneumoniae*. This pathogen is responsible for causing an estimated 1.6 million deaths annually. WHO reported these figures in 2005 [99]. Before introducing pneumococcal vaccines during the 1990s, around 63,000 cases (children only) were reported on average each year in the USA [100]. In parallel to this, the resistance to drugs like penicillin and other antibiotics was also reported to be developing, with invasive pneumococci becoming resistant to three or more classes of drugs [101,102]. Expectedly, the vaccine’s introduction was a tremendous success with more than 90% efficacy against the invasive pneumococcal disease (IPD) in children less than five years of age. Reportedly, the vaccine in parallel to preventing the disease also reduced the bacterial colonization in children. This, in the end, contributed towards the herd immunity in the subjects who were not targeted for immunizations [103].

## 10. Inhibition of Quorum Sensing

Microbes communicate with each other by using signal molecules to exchange information, known as quorum sensing. Microorganisms use this information to initiate the infection and expressed the pathogenicity in eukaryotes through regulation. The main issue from quorum sensing is the formulation of biofilms, stimulation of the efflux pump, which increases the bacterial antibiotic resistance [104]. Pathogens occupied the host by forming colonies active the quorum sensing, resulting in the production of virulence factors and biofilms. This suggests that to break the signaling, it is recommended to break this bacterial conversation by utilizing the anti-quorum sensing agents and increase the susceptibility of pathogens to antibiotics and host immunity. To tackle this issue, quorum sensing inhibition strategies are introduced from diverse origins, which have shown potential as a therapeutic target, including receptor inactivation, signal degradation, blocking of signals by an antibody, and inhibiting the signal synthesis [105].

## 11. Other Molecular Mechanisms

### 11.1. Next-Generation Sequencing and Antimicrobial Peptide Prediction

There is a need to understand the susceptibility of an infectious agent and host resistance mechanism for the development of innovative approaches to prevent or treat human infectious ailments [106]. Technologies like next-generation sequencing revolutionized the world of science. They opened the door for the researcher to understand different organisms’ physiological responses through genomics and transcriptomes at high throughput. As a result, new tools are introduced to design novel antimicrobials [107]. Antimicrobial peptides (AMPs) are tools to fight antimicrobial resistance, which are constantly searched for in different organisms.

The venom of scorpion *Hetermetrus petersii* consists of four antimicrobial and cytosolic peptides as shown by platform 454 sequencing [108]. A few genes consisting of those encoding AMPs are down-regulated as specified by the 454-analysis due to AcMNPV infection in *Spodoptera exigua* larvae. For perspective, different techniques are revealed in various organisms. New viewpoints are offered for AMP mode of action due to the linkage between transcriptome and proteomics technologies. In the bivalve mollusk *Ruditapes philippinarum,* the identification of 36 AMP sequences is due to the use of 454 platforms [109]. In American dog tick being affected by various microbes, the transcriptome analysis helped identify new defense mechanisms in the response transcripts of the Arachnids immune system [110]. Because of increased diversity in organisms and different tissues, a variety of novel AMPs obtained from biodiversity opens a new area for research purposes.

Molecular surveillance using whole-genome sequencing (WGS) can be a valuable addition to AMR’s phenotypic surveillance [111]. With the advent of WGS technology, it is now possible to determine and evaluate the entire genome sequence of microorganisms at low costs in a limited time, making it an ideal tool for bacterial antimicrobial resistance surveillance. By providing definitive genotype information, WGS offers the highest practical resolution for characterizing an individual microbe. This includes the full complement of resistance determinants, including resistance to compounds not routinely tested phenotypically. WGS can also differentiate bacteria that have identical resistance patterns caused by different mechanisms. What makes WGS ground-breaking is that it can help researchers predict antimicrobial resistance more efficiently and offer valuable information to augment or supplant the phenotypic approaches in clinical decision making. However, its cost-related issues and complexity, WGS is currently carried out in high-income countries. Establishing WGS as a surveillance tool could be very important in producing an accurate global picture and informing the national and international action plans against AMR. The online database developed named as BacWGSTdb 2.0 which provides a quick and convenient tool for monitoring the antimicrobial resistance and pioneering the movement of WGS from proof-of-concept studies to routine use in the clinical microbiology laboratory [112]. The mentioned database is designed for clinical microbiologists, hospital epidemiologists, and clinicians. BacWGSTdb offers a convenient and rapid platform for users worldwide to address the clinical issues related to antimicrobial resistance [113].

### 11.2. Prediction of Antimicrobial Peptide from DNA/RNA Library: Antimicrobial Peptides Search Tools

The native and acquired immune responses of organisms have a direct relationship with the antimicrobial peptides, and their ability to destroy microorganisms that are resistant to a variety of antibiotics has been an area of interest to the pharmaceutical field. In this regard, keys to search and generate antimicrobial peptides have opened new opportunities in new drugs’ research. Development in bioinformatics has given way to a routine search in ESTs’ databases in plants via defensins and a testing validation such as antimicrobial testing [114]. One of the primary databases of the antimicrobial peptides includes APD2 (antimicrobial peptide database second version) that permits users to research peptide families and modified peptides [115]. To obtain necessary information regarding peptides like total charge or the rate of hydrophobicity along the alignment of sequences, APD2 gives an opportunity to calculate AMPs [116]. Such information could be related to the data about hydrophobic phenomena predicted via web-program known as HydroMcalc [117,118]. Thus, making possible antimicrobial peptides’ identification. Along with APD, CAMP (Collection of Antimicrobial Peptides) is a similar AMP database [119]. These techniques refer to the sequencing, protein biological effect, source organisms’ taxonomy, MIC identifying target organisms, peptides’ hemolysis process, and relation with the external databases like SwissProt, PDB, PubMed, and the NCBI Taxonomy [120]. A web-server iAMP-2L [121] is used for the identification of sequences that are uncharacterized like antimicrobial. When a sequence is predicted as antimicrobial, the server shows which category (antibacterial, anticancer, antifungal, anti-HIV, and anti-viral) peptide is related. Peptides are unstable molecules and are categorized into several classes and families [122]. Such fundamentals brace how these novel techniques can disclose many novel drugs and biologically active compounds. For the development of such a research field, keys for the modeling of bioinformatics are necessary [123].

## 12. Conclusions

Antimicrobial resistance, especially in the ESKAPE pathogen, is an intricate multifactorial process. Many factors contribute to the increase in resistance to antimicrobials in these pathogens. Studies have concluded that there are diverse genetic processes involved in the development of resistance in ESKAPE pathogens. There are different strategies developed to evade multi-drug resistance in ESKAPE pathogens. Novel antimicrobials, ranging from nanomaterials to antimicrobial peptides, have been developed to overcome the challenges of antimicrobial resistance. 1.7 million people are affected Although many new strategies show promising results in few pathogens, clinical microbiologists should keep their keen eyes on an even slight increase in the minimum inhibitory concentration of antimicrobials that predicts uncontrollable resistance. Moreover, the mechanism by which antibiotics imply selective pressure is poorly understood. There is an utter need to make specific suggestions that will help improve the studies related to natural selection of these organisms on antibiotics. These types of understanding play a fundamental role in designing a rational dogma of antibiotic practices to maximize existing antibiotics’ lifespan and minimize the influence of resilient infections.

## Figures and Tables

**Table 1:** Narrative of pathogenic bacterial strains (ESKAPE) that instigated nosocomial infection [49].

Bacterial Strain	Gram Staining Type	Resistance Type	Antibiotics	Treatment Option	Resistance Level
*Acinetobacter*	Negative	Multidrug	Ceftazidime, aminoglycoside, fluoroquinolones, carbapenems	*Carbapenems*, b*-Lactamase inhibitors*, *Tigecycline*, *Aminoglycosides*, *Polymyxin therapy*, *Synergy*, and *combination therapy*	High level
*E. coli*	Negative	Multidrug	Cephalosporins (ESBL-producers), fluoroquinolones, aminoglycosides	GyrB/ParE programme, EV-035	High level
*K. pneumoniae*	Negative	Multidrug	Cephalosporins (ESBL-producers), fluoroquinolones, aminoglycosides, carbapenems	POL7080 and ACHN-975 compounds	High level
*P. aeruginosa*	Negative	Multidrug	Piperacillin/tazobactam, ceftazidime, ciprofloxacin, aminoglycosides, carbapenems	POL7080 and ACHN-975 compounds	High level
*Enterococcus spp.*	Positive	Multidrug	Ampicillin, aminoglycosides, glycopeptides	RX-04 lead series, 50S ribosomal subunit; inhibit translation by stabilizing a distorted mode of P-tRNA binding	High level
*S. aureus*	Positive,	Multidrug	β-lactam antibiotics (except new anti- methicillin-resistant *S. aureus* cephalosporins), macrolides, fluoroquinolones, aminoglycosides	RX-04 lead series, 50S ribosomal subunit; inhibit translation by stabilizing a distorted mode of P-tRNA binding	High level

**Table 2:** Types of antimicrobial resistance at the cellular level.

Resistance	Proposed Mechanism	Examples	Ref.
Inactivation of Drug	Use of hydrolysis or modification	b-lactamase for b-lactam resistance, acetyltransferases for aminoglycoside resistance	[53,54]
Alteration of Target	Reduction of binding affinity to the drug by bypassing the drug target	DNA gyrase mutation for fluoroquinolone resistance	[55]
Drug influx Reduction	By decreasing permeability	Gram-negative outer membrane	[56]
Extrusion of Drug	Efflux pumps	Accessory membrane fusion proteins	[57]
Horizontal gene transfer	By resistance determinants from other microorganisms		[58]

**Table 3:** Mechanism of bactericidal activity of Nanoparticles and synergic effect of antibiotic-conjugated metal oxide nanoparticles against ESKAPE Pathogen.

Nanoparticles (NP)	Mode of Action/Mechanism of Nanoparticles Against ESKAPE Pathogens	Antibiotic Used	Microorganism	Synergic Effects (Antibiotics-Nanoparticles)	Ref.
AgNPs	Damage the bacterial cell membrane and disrupt the activity of membranous enzymes. Cell wall distraction by cell DNA was condensed to a tension state and could have lost its replicating abilities	Doxycycline	*K. pneumoniae*	Observed	[68]
		Gentamicin and Neomycin	*S. aureus*	AgNPs + Gentamicin showed resistance in 50% strains while AgNPs + Neomycin showed synergy 45% of the strains.	[69]
			*E. coli*, *S. aureus*	Observed increase in activity was such that Erythromycin showed 18.9.6%, Kanamycin = 27.9.3%, Chloramphenicol = 18.1.3%, and Ampicillin = 74.8.9%	[69]
		β-Lactam, cefotaxime	*E. coli*, *S. aureus*	Synergistic increase in activity was such that 17.2%, 13.5% for *E. coli* and *S. aureus*, respectively	[70]
		Ampicillin, chloramphenicol, and kanamycin	*S. aureus*, *E. coli*, and *P. aeruginosa*	Synergistic effects observed	[71]
		Beta-lactam: cephem	*S. aureus*	Cephalothin and cefazolin showed a 30% increase in activity when used in combination with 20 μg/ mL AgNPs against *Micrococcus luteus, and Bacillus subtilis*	[72]
AuNPs	Disturb membrane potential by inhibiting ATPase activities; inhibit the subunit of the ribosome from binding tRNA. Cellular death induced by gold nanoparticles do not include reactive oxygen species-based mechanisms	Ampicillin, streptomycin, and kanamycin	*E. coli* and *S. aureus*	15%, 12%, and 34% increase in inhibition zone for *E. coli* with A/S/K+Au, respectively; 20%, 109%, and 18% increase in inhibition zone for *M. luteus* A/S/K+AuNPs, respectively; 12% and 34% increase in inhibition zone for *S. aureus* with A/ K+AuNPs, respectively	[73]
		Beta lactams: cefaclor	*S. aureus* and *E. coli*	MICs of cefaclor reduced gold nanoparticles were 10 mg/mL and 100 mg/mL for *S. aureus* and *E. coli,* respectively	[74]
ZnONPs	Interactions between reactive oxygen species and membrane proteins result in cell damage. ZnO-NPs disrupt bacterial cell membrane integrity, reduce cell surface hydrophobicity, and down-regulate the transcription of oxidative stress-resistance genes in bacteria	Ceftriaxone	*E. coli*	Synergistic antibacterial effects against *E. coli* have been observed by ZnO nanorods with ceftriaxone	[75]
		Ciprofloxacin	*S. aureus* and *E. coli*	Increase in inhibition zones in *S. aureus* = 27% and 22% in *E. coli* when ciprofloxacin and ZnONPs were applied in synergism	[76]
		Beta lactams, aminoglycosides, and azolides	*S. aureus*	The highest increase was observed for penicillin G and amikacin, i.e., 10 mm increase in the zone of inhibition, whereas for clarithromycin, a 2 mm increase had been observed	[77]
TiO_2_NPs	Electrostatic interaction between TiO_2_ NPs and the bacterial cell surface results in suppression of cell division, degradation of the cell wall and cytoplasmic membrane due to the production of reactive oxygen species such as hydroxyl radicals and hydrogen peroxide	Penicillin G, amikacin, cephalexin, cefotaxime	MRSA	10 mm increase in zone size. TiO_2_ nanoparticles significantly improved antibiotic efficacy against *S. aureus* when combined with beta-lactams, cephalosporins, and aminoglycosides	[78]
Fe_3_O_4_NPs	Generation of reactive oxygen species from the disruption of the electronic transport chains owing to the resilient affinity of the iron-based nanoparticles for the cell membrane. Reactive oxygen species generated by Fe3O4 nanoparticles kill bacteria without harming non-bacterial cells	Streptomycin	*S. aureus*, *E. coli*, and *P. aeruginosa*	Zones of inhibition at concentrations (10, 20, 40, and 80): *S. aureus* (15 mm, 14 mm, 17 mm, 20 mm), *E. coli* (12 mm, 14 mm, 15 mm, 17 mm), *P. aeruginosa* (13 mm, 14 mm, 15 mm, 18 mm)	[79,80,81]
		Kanamycin and rifampicin	*E. coli* and *S. aureus*	Kanamycin formed an inhibition zone against both, whereas rifampicin formed an inhibitory zone against *S. aureus* only	[81]
		Amoxicillin	*E. coli* and *S. aureus*	A total of 9.9% and 8.9% increase in inhibitory effect observed in the presence of Cu NPs for *E. coli* and *S. aureus,* respectively	[80]
CuNPs	Generation of reactive oxygen species, lipid peroxidation, protein oxidation, and DNA degradation. Cu2+ ions released from nanoparticles penetrate bacterial cells and are subsequently oxidized intracellularly	Amikacin, ciprofloxacin, gentamicin, norfloxacin	*E. coli*, *P. aeruginosa*, *Klebsiella spp. S. aureus*	At 60 mg/mL, 18 mm for *E. coli*, 16 mm for *Klebsiella*	[82]
BiNPs	Production of reactive oxygen species	Ciprofloxacin, norfloxacin, tetracycline, and metronidazole	*K. pneumoniae*	A synergistic effect was observed between all antibiotics and BiNPs.	[83]
		Cefotaxime, ampicillin, ceftriaxone, cefepime	*E. coli*, *K. pneumoniae*, and *P.aeruginosa*	Significant decrease in MIC decrease with cefotaxime and ZnO NPs against *K. pneumoniae* (85.7%), *P. aeruginosa* (70%), and *E. coli* (50%) has been observed. Meanwhile, a decrease in MIC due to ZnO NP with other antibiotics has been observed.	[84]
		Norfloxacin, Ofloxacin, and Cephalexin	*P. aeruginosa*, *E. coli*	Significant increase in inhibition zone of antibiotics with ZnONPshave been observed against all isolates.	[67]

## Abbreviations

ESKAPE	*Enterococcus faecium, Staphylococcus aureus, Klebsiella pneumoniae, Acinetobacter baumannii, Pseudomonas aeruginosa*, and *Enterobacter species*
AMP	Antimicrobial peptides
AMU	Antimicrobial usage
ARGs	Antimicrobial resistant genes
ESBL	Extended-spectrum b-lactamases
HAIs	Hospital-acquired infections
Hib	*Haemophilus influenzae* type b
HGT	Horizontal gene transfer
IPD	Invasive pneumococcal disease
MDR	Multidrug resistant
MRSA	Methicillin-resistance *Staphylococcus aureus*
NLRs	NOD-like receptors
PRRs	Recognition receptors
TLRs	Toll-like receptors
WGS	Whole genome sequencing
XDR	Extensively drug-resistant
